# Electrochemical polishing of additively manufactured titanium alloys: a mechanism-to-parameter framework for process design

**DOI:** 10.3389/fchem.2026.1906332

**Published:** 2026-08-24

**Authors:** Shin Young Oh, Jiwon Kim

**Affiliations:** Advanced Materials Research Center, Institute for Advanced Engineering Chungcheong Campus, Chungju, Republic of Korea

**Keywords:** additive manufacturing, current distribution, electrochemical polishing, mass transport, process window, Ti-6Al-4V, titanium

## Abstract

Additive manufacturing (AM) has expanded the use of titanium and its alloys in aerospace, biomedical, marine, and automotive components, but the rapid melting–solidification cycles inherent to AM leave characteristic surface defects—balling, partially melted particles, scan-track ridges, and near-surface porosity—that make post-process finishing essential. Electrochemical polishing (ECP) is a leading candidate because material is removed by anodic dissolution, allowing treatment of geometrically complex surfaces inaccessible to mechanical methods. The literature available to newcomers remains divided: classical electropolishing theory addresses idealized surfaces prior to AM, while recent AM reviews focus empirically on electrolytes and roughness outcomes rather than current-distribution and mass-transport physics. Consequently, researchers often reproduce published conditions as empirical recipes, relying on trial-and-error optimization. This review is therefore organized not as a chronological survey but as a mechanism-to-parameter design framework for engineers adopting ECP for Ti and Ti-6Al-4V. It (i) relates classical current-distribution theory (primary, secondary, and tertiary distributions; the Wagner number (Wa)) and salt-film and acceptor-based mass-transport mechanisms to macro- and micro-smoothing of AM Ti surfaces and to the diffusion-limited plateau of the J–V curve that defines the practical process window; (ii) decomposes electrolytes into functional units—solvent, supporting electrolyte, acid, and additive—so that composition becomes a set of design rules rather than a list of recipes; (iii) consolidates acid-based, organic, ionic-liquid, and deep-eutectic systems, and AM and wrought substrates, into a unified condition database; and (iv) positions finite-element (COMSOL and others) prediction of fields, current-density distributions, and material removal as a means of narrowing the experimental search space. Viewed through this framework, the diversity of reported ECP conditions converges on one objective: forming and sustaining a stable, mass-transport-controlled interfacial layer. Viscosity, additive chemistry, agitation, temperature, and waveform are not independent variables but alternative routes to controlling the diffusion layer, and specimen geometry sets how much geometry-driven current concentration can contribute to leveling. Framed this way, condition selection becomes a reasoned choice of where to sit within the process window rather than searching across a catalogue of recipes—the basis on which ECP can be transferred from coupon-level demonstrations to component-scale Ti parts.

## Overview of electrochemical polishing in additive manufacturing

In recent years, titanium (Ti) and its alloys (e.g., Ti-6Al-4V, Ti-6Al-7Nb) have attracted significant attention across various industries, including aerospace, marine, medical, and automotive because of their light weight, high strength, excellent corrosion resistance, and environmental friendliness (Acquesta and Monetta, 2021; Chaghazardi and Wüthrich, 2022; Han and Fang, 2019; Kim et al., 2015; Lausmaa, 2001; Liu et al., 2006; Zhang et al., 2020). Especially with the advancement of additive manufacturing (AM), also known as 3D printing, the applicability of Ti-based powder materials in a wide range of industrial manufacturing fields has been extensively explored due to the advantages of a simple process compared to conventional casting methods with superior mechanical properties (Acquesta and Monetta, 2020; 2023; Jiang et al., 2022; Mu et al., 2023; Roy et al., 2024).

**FIGURE 1:**
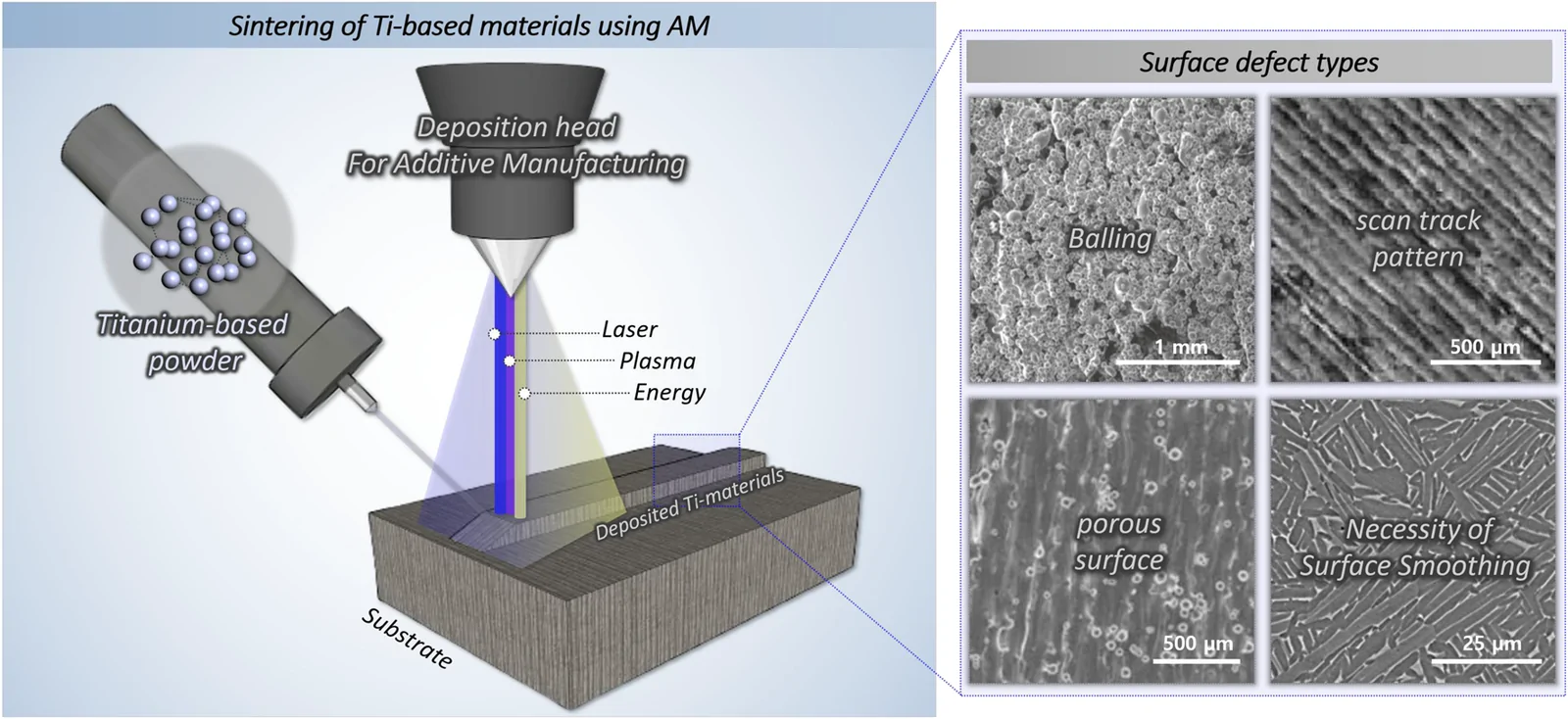
(Left) Schematic illustration of a directed energy deposition process using powder feedstock and a focused energy source. (Right) Surface defect types—such as balling, scan track patterns, and porous surfaces—resulting from the AM process are shown (Adapted from Mhlanga et al., 2024; Roy et al., 2024; Tsoeunyane et al., 2022; Xiao et al., 2024).

Figure 1 (left) shows a schematic illustration of the AM process using Ti-based powder as the source material (Mhlanga et al., 2024; Roy et al., 2024; Tsoeunyane et al., 2022; Xiao et al., 2024). Among fusion-based metal additive manufacturing processes, powder bed fusion (PBF) and directed energy deposition (DED) represent two major categories (Acquesta and Monetta, 2021; Mu et al., 2023; Sun et al., 2017). In PBF, a thin layer of metal powder is spread over a build platform and selectively melted using a focused energy source (Acquesta et al., 2023; Acquesta and Monetta, 2023; Pathi et al., 2021; Xiao et al., 2024). Depending on the energy source, PBF is classified as laser powder bed fusion (L-PBF), commonly referred to as selective laser melting (SLM), or electron beam powder bed fusion (EB-PBF), also known as electron beam melting (EBM). In contrast, DED directly feeds powder or wire into a melt pool generated by a laser, electron beam, or electric arc while depositing the molten material onto a substrate. Direct laser deposition (DLD) and arc-based processes such as wire arc additive manufacturing (WAAM) belong to this category. These fusion-based additive manufacturing processes involve rapid and repeated melting–solidification cycles, which may generate characteristic surface irregularities and defects, such as partially melted particles, scan-track ridges, balling, and near-surface porosity, depending on the process type and operating conditions (Chaghazardi and Wüthrich, 2022; Mhlanga et al., 2024; Mu et al., 2023; Tyagi et al., 2025).

As illustrated in Figure 1 (right), surface defects such as balling, scan track patterns, and porosity are inevitably formed, leading to a non-uniform surface morphology. Such surface irregularities are not unique to AM; comparable roughening arises in other thermal and joining processes such as fusion and friction-stir welding, where the resulting surfaces often benefit from post-processing before service (Gangwar and Ramulu, 2018; Reyaz et al., 2024; 2025; Reyaz and Sinha, 2025). In this context, the defect-prone morphology of AM Ti alloys requires post-processing to achieve the surface quality demanded by their intended applications. Among the available finishing routes, mechanical polishing is relatively simple but poorly suited to geometrically complex surfaces, whereas chemical polishing can treat complex geometries but often suffers from slow reaction rates and environmental and safety concerns associated with strongly acidic or alkaline solutions (Acquesta and Monetta, 2021; 2023; Chaghazardi and Wüthrich, 2022; Mu et al., 2023; Zhang et al., 2020). ECP, which smooths and brightens metal surfaces by promoting preferential anodic dissolution at surface protrusions, has therefore emerged as a promising finishing method for complex AM Ti components (Han and Fang, 2019; Tsoeunyane et al., 2022; Yang et al., 2017). Despite this potential, selecting suitable ECP conditions for AM Ti alloys remains difficult because the mechanistic basis of electropolishing and the application-oriented AM literature have largely developed as separate bodies of knowledge (Kołkowska et al., 2024). Classical electropolishing theory predates AM and was developed largely for idealized model surfaces, establishing the coupling between current distribution, mass transport, and surface leveling (Han and Fang, 2019; Landolt, 1987; Wagner, 1954). Recent AM-oriented reviews, in turn, have surveyed electrolytes, operating conditions, and roughness outcomes for AM parts, with the connection back to that physics falling outside their intended scope. Table 1 summarizes the scope of these reviews (Acquesta and Monetta, 2020; Chaghazardi and Wüthrich, 2022).

**TABLE 1:** Scope of previous reviews on electropolishing relevant to AM Ti alloys, and the position of the present review.

Review	Materials in scope	Substrate	Mechanistic framework	Electrolyte treatment	Predictive modeling
Landolt (1987)	Metals in general	Conventional and model surfaces	Macro-/micro-smoothing distinction; current-distribution theory; Wagner’s leveling analysis; mass-transport-controlled dissolution	General physicochemical principles	Analytical and numerical current-distribution methods
Han and Fang (2019)	Metals in general; Ti treated briefly	Mainly conventionally processed	Viscous-film, salt-film and acceptor mechanisms; four-region J–V behavior; process variables	Grouped by electrolyte type (aqueous acid, organic)	Outside scope
Acquesta and Monetta (2021)	Ti and Ti-6Al-4V	AM	Smoothing and brightening described phenomenologically; mechanistic theory outside scope	Compiled per study as reported conditions	Outside scope
Chaghazardi and Wüthrich (2022)	AM metals in general	AM	Current-distribution and geometric effects noted; focus on AM-specific challenges and outlook	Grouped by metal system	Identified as a future direction
Acquesta and Monetta (2023)	Multiple alloy systems	AM	Anodic leveling and primary current distribution noted; focus on electrolyte chemistry	Grouped by chemistry class; green, Deep eutectic solvents (DES)- and Ionic liquids (IL)-based systems	Identified as future scope
This review	Ti and Ti-6Al-4V	AM and wrought in a single frame	Primary, secondary, tertiary current distributions and the Wa coupled with salt-film/acceptor mass transport; mapped onto macro-/micro-smoothing and region III	Decomposed into functional units—solvent, supporting electrolyte, acid, additive—as design rules; acid-, organic-, IL- and DES-based systems consolidated	FEM/COMSOL prediction of field, current density, mass transport, and material removal, positioned as a means of contracting the experimental parameter space

Each review focuses on a different aspect of the problem, but none fully connects the underlying mechanisms to their practical use in AM. As a result, newcomers are often given only the reported process conditions without a clear explanation of why they work, forcing them to repeat the optimization process from the beginning.

**SCHEME 1:**
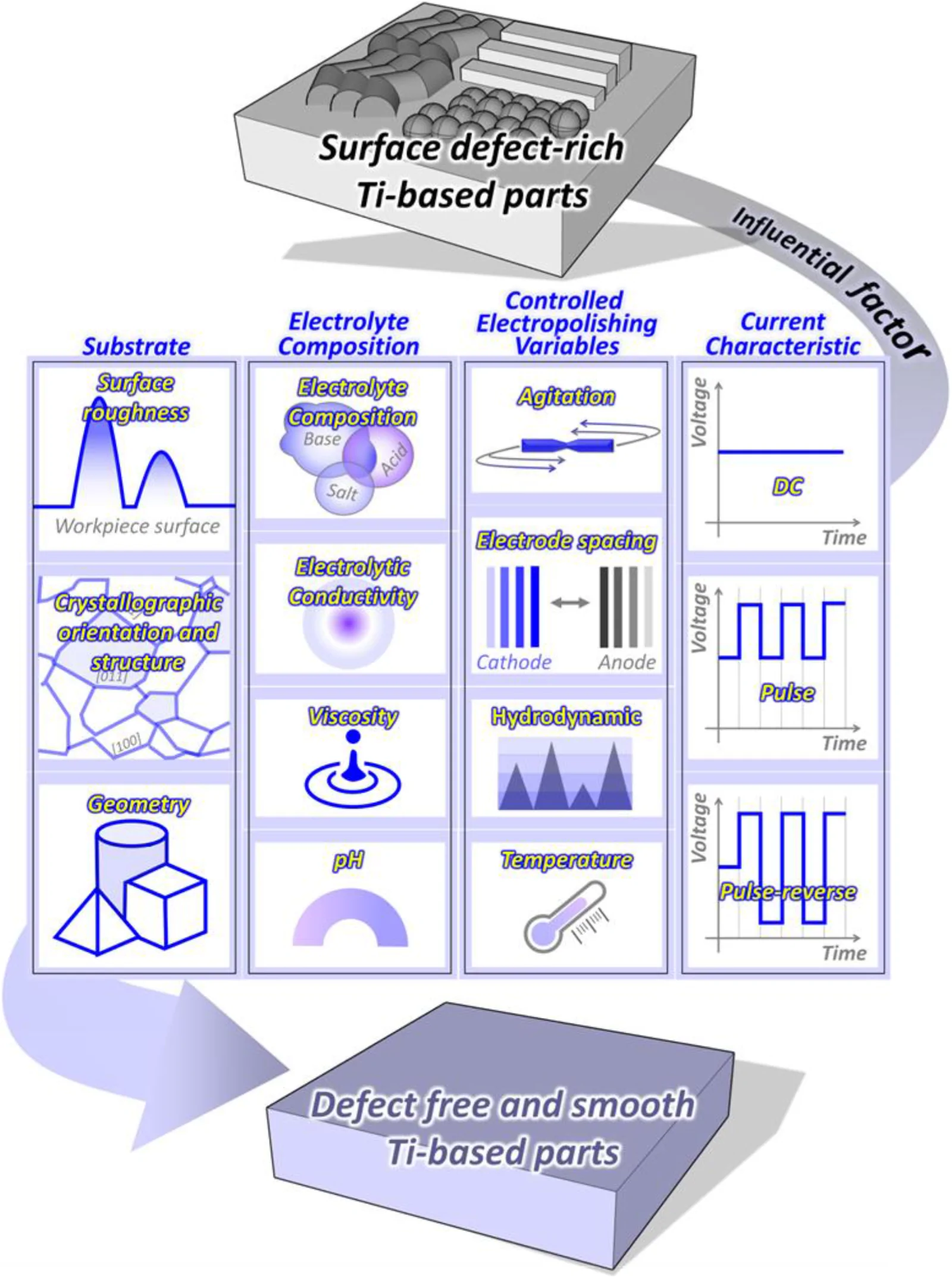
Schematic overview of the key factors influencing electrochemical polishing of Ti-based parts, including substrate characteristics, electrolyte composition, controlled electropolishing variables, and current characteristics, and their roles in achieving defect-free and smooth surfaces.

This review is therefore organized not as a chronological survey but as a mechanism-to-parameter design framework for materials and mechanical engineers adopting ECP for Ti and Ti-6Al-4V. Its contributions are fourfold. First, classical current-distribution theory—primary, secondary, and tertiary distributions and the Wa—is coupled with salt-film and acceptor-based mass-transport mechanisms and mapped directly onto the macro- and micro-smoothing of AM Ti surfaces and onto the diffusion-limited plateau (region III) of the J–V curve that defines the practical process window. Second, electrolyte formulations are decomposed into functional units—solvent, supporting electrolyte, acid, and additive—so that composition can be read as a set of design rules rather than a list of recipes (Table 2). Third, acid-based, organic, ionic-liquid (ILs), and deep-eutectic systems (DESs), together with AM and wrought substrates, are consolidated into a unified condition database for Ti and Ti-6Al-4V (Table 3). Fourth, finite-element modeling (COMSOL) is positioned not as a reporting exercise but as a means of contracting the experimental parameter space before an electrolyte is ever mixed. The review proceeds from the fundamental principles of chemical, electroless, and electrolytic polishing, through the three parameter families that govern ECP—specimen, electrochemical parameter, and electrolyte—to industrial applications and simulation-assisted process design. The overall framework linking substrate characteristics, electrolyte composition, controlled electropolishing variables, and current characteristics to the surface quality of Ti-based parts is summarized in Scheme 1.

**TABLE 2:** Composition of a typical organic-based Ti electropolishing solution.

Organic-based Media = Solvent + Supporting electrolyte + Acid (mix or single) + Additives			
Type	Content (%)	Function	Example
Solvent	40–95	Base solvent of the electrolyte (e.g., organic solvents for Ti) Control viscosity of an electrolyte	Ethylene glycol, ethanol, Methanol
Supporting electrolyte	5–15	Minimize the IR drop	CaCl_2_, NaCl
Acid	10–30	Participates in dissolution reaction, removes metal oxides, adjusts pH	H_2_SO_4_, HCl, HClO_4_, CH_3_COOH
Additives	∼1	Enhances electrolyte polarity	H_2_O
	0–30	Viscosity modifier, diffusion controller, passivation breakdown, complexing agent, water scavengers, wetting agents and surfactants	Fluoride donors, carboxylic and phosphonic ligands, acetic anhydride, sodium dodecyl sulfate (SDS), polyethylene glycol (PEG)

**TABLE 3:** Summary of ECP conditions for Ti and Ti-6Al-4V alloy, organized by the functional decomposition of Table 2.

Titanium (ti)													
Electrolyte composition						Operating conditions				Key results			​
No.	Electrolyte type	Electrolyte				Temperature (°C)	Agitation (speed)	Applied voltage (V)/current density (A/cm2)	Polishing time (min)	Surface outcome	Ra initial (nm)	Ra final (nm)	Reference
		Solvent	Supporting electrolyte	Acid	Additive								
1	Organic-based	Ethylene glycol	ChCl	-	H_2_O	35	<80 rpm	16 V	15	Clean and flat	-	514	Hwang and Kim (2025)
2		Ethylene glycol	NaCl (1 M)	-	Ethanol (15–25 vol%)	-	proper agitation	20 V	5	-	1,460	220	Mahardika et al. (2021)
3		Propylene glycol	ChCl	-	-	60	continuous stirring	10 V	40	Mirror-like surface	455.6	37.92	Karim (2021)
4		Ethylene glycol	NaCl	-	Ethanol (0–30 vol%)	20 ± 3	100 rpm	20 V	50	Mirror-like and smooth surface	-	2.341	Kim et al. (2015)
5		Methanol	-	Perchloric acid	Ethylene glycol	0 ± 1	300–1,300 rpm	20 V	1–5	Mirror-like finish	-	2.53	Peighambardoust and Nasirpouri (2014)
6		Ethanol	-	H_2_SO_4_	H_2_O (0–5 vol%)	25 ± 1	100 rpm	6 V (vs. Ag/AgCl)	-	Defect free surface, improvement of the brightening	-	1.85	Huang et al. (2011)
7		Ethanol, Butanol,Methanol	ChCl	-	-	20	-	2, 1.2 V	40	Free from defects	120.5	0.10	Karim et al. (2021)
8		Ethylene glycol	ChCl	-	-	20	Continuous stirring	6–10 V	30	Apparent mirror-like finished surface	118.8	5.7	Karim et al. (2020)
9		TMHA-Tf_2_N ionic liquid	-	-	H_2_O	50	Proper agitation	2–9 V vs. I^3-^/I^−^	-	Mirror-like surface with brightness	-	-	Uda et al. (2011)
10	Aqueous-based	Methanol, C_2_H_6_O_2_ monobutylether	-	Perchloric acid	HF	15	200 rpm	52 V, 1 min followed 28 V, 13 min	14	Clear surface	-	1.966 (Rz)	Chen et al. (2005)

Titanium alloy: Ti-6Al-4V													
No.	Electrolyte type	Electrolyte				Temperature (°C)	Agitation (speed)	Voltage(V) /Current density (A/cm^2^)	Polishing time	Surface outcome	Ra initial (nm)	Ra final (nm)	References
		Solvent	Supporting electrolyte	Acid	Additive								
11	Organic-based	Methanol	-	Sulfuric acid	-	−20	Proper agitation	10–15 V	10	Glossy, smooth, reflective metallic surface	-	0.09	Kołkowska et al. (2024)
12		Urea	ZnCl_2_	-	-	90	260 rpm	0.6 A cm^−2^	10	Improve surface hydrophobicity, corrosion resistance	<12,000	>3,000	Tang et al. (2024)
13		Ethylene glycol	NaCl	-	Ethanol	25	0–1,150 rpm	20 V	15–60	Glossy surface	50,900	890 ± 100	Acquesta et al. (2023)
14	​	Triethanolamine + ethanol	NaF + NH_4_F	Perchloric acid + Oxalic acid	-	30 ± 2	400 rpm	0.2 A cm^-2^	30	Reduces the average surface roughness, waviness	45,220	9,310	Tsoeunyane et al. (2022)
15		Ethylene glycol	NaCl	-	-	30–40	Proper agitation	20 V	15	Uniform surface	-	-	Jiang et al. (2022)
16		Ethanol	-	Acetic acid + perchloric acid	Oleic acid	4	-	20 V	7	Highest uniformity of the surface finish	27,170	7,570	Nan Kuo et al. (2022)
17		Methanol	-	Perchloric acid	Ethylene glycol	5–40	-	20 V/1–4 A cm^-2^	2–10	Flattening surface	-	<400	Wang et al. (2021)
18		Ethylene glycol	MgCl_2_	-	H_2_O	25 ± 0.1	500 rpm	0.5 A cm^−2^	5–15	Clean and formation of TiO_2_ layer	8,330	1,090	Zhang et al. (2020)
19	​	Acetic acid + ethanol	-	Perchloric acid	-	-	800 rpm	0.147/0.294/0.442 A cm^-2^	20	Smooth surface	24,000	15,000/10,000/5,000	Wu et al. (2019)
20		Ethylene glycol + isopropyl alcohol	AlCl_3_ ZnCl_2_	-	-	30	Proper agitation	70 V	30	Homogeneous surface finish	6,830	540	Benedetti et al. (2017)
21	Aqueous-based	Glacial acetic acid	-	Perchloric acid	H_2_O	30	-	0.3 A cm^-2^	0–20	Smoothing of prominent parts	7,000	1,000	Zhang et al. (2018)
22		Glacial acetic acid	-	Perchloric acid Acetic acid	-	23	100 rpm	0.24 A cm^-2^	27	Uniform surface	-	-	Urlea and Brailovski (2017)
23		Acetic acid	-	HF; HNO_3_	-	25	-	55 V/0.3 A	5	Smoothing of prominent parts	6,000	4,000	Longhitano et al. (2015)
24		Acetic acid (glacial, 900 mL/L)	-	Perchloric acid	Oxalic acid Acetic acid	Low temperature	-	25 V	20	Smoothness ·Uniformity · Defect reduction	8,470	1,090	Chen et al. (2022)

### Fundamental principles of electrochemical polishing

**FIGURE 2:**
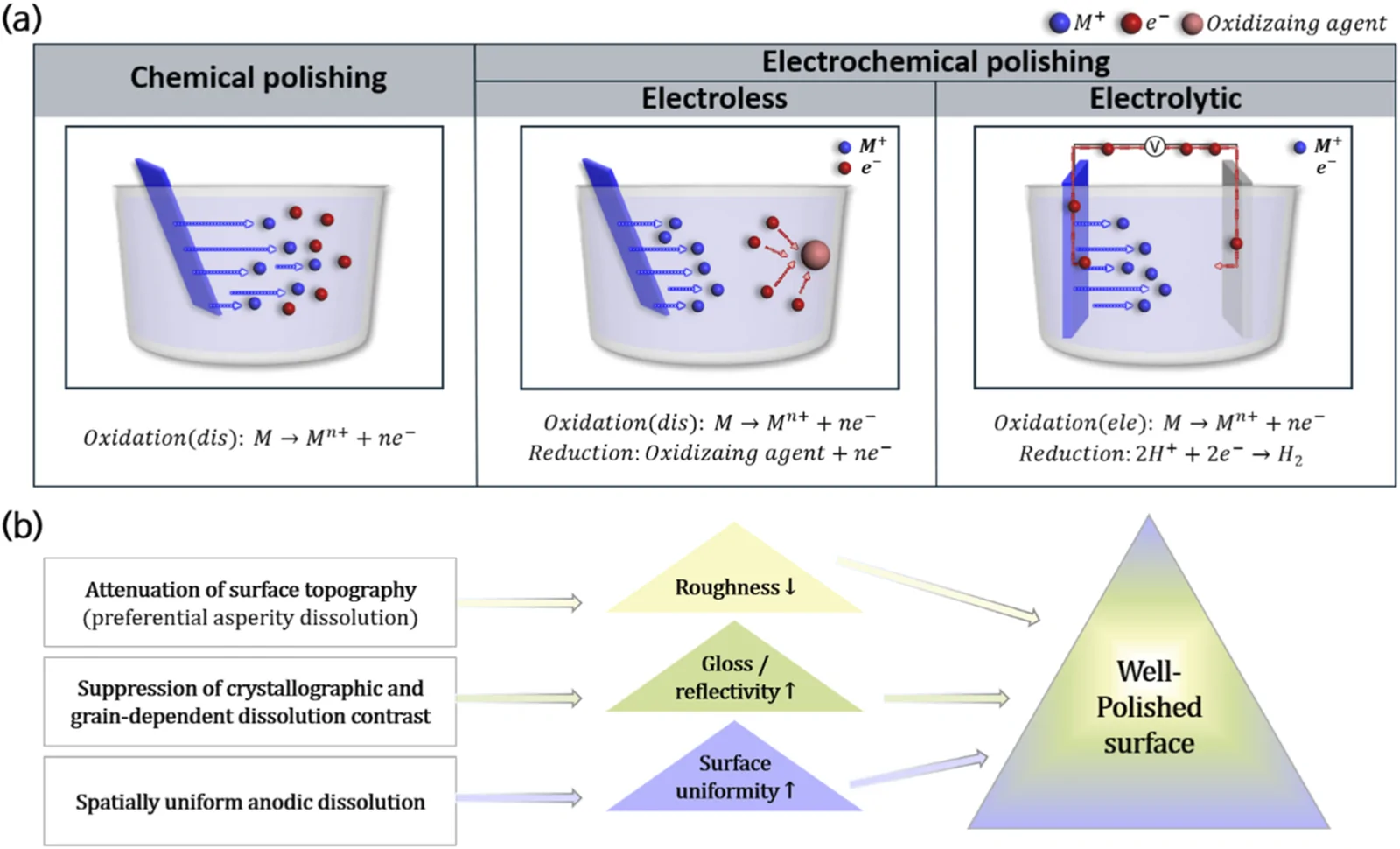
**(a)** Schematic illustration of chemical and electrochemical polishing, including their reactions. Electrochemical polishing is categorized into electroless and electrolytic processes. **(b)** Schematic linking the three dominant electropolishing mechanisms to their respective outcomes and the resulting well-polished surface.

The ultimate objective of polishing is to produce a flat surface by minimizing roughness and to achieve a smooth, glossy finish without introducing visible damage at the crystal or grain level. Chemically based routes—chemical, electroless, and electrochemical polishing—are attractive for AM parts because they impose few structural constraints on complex geometries (Acquesta and Monetta, 2023; Mu et al., 2023; Tsoeunyane et al., 2022). Schematic illustrations and representative chemical reactions for the three proposed polishing methods are presented in Figure 2a. In chemical polishing, the specimen is directly immersed in a polishing bath, where the acidic or basic medium of the electrolyte induces surface oxidation–reduction reactions. These reactions dissociate the metal (M^0^) into metal ions, leading to surface dissolution and thereby achieving polishing in Equation 1. (Chaghazardi and Wüthrich, 2022; Lane et al., 2015; Lebedeva et al., 2021; Tailor et al., 2015; Tyagi et al., 2019; Yang et al., 2017) However, as dissolution proceeds, the concentration of dissolved metal species in the bath approaches its solubility limit, and the polishing reaction slows and eventually ceases unless the solution is replaced (Jacquet, 1956; Peighambardoust and Nasirpouri, 2014; Steigerwald et al., 1995; Wu et al., 2019).

$$Oxidation:{Ti}^{0}\to {Ti}^{4+}+{4e}^{-}$$ (1)

Electrochemical approaches can be further categorized into electroless and electrolytic (Chaghazardi and Wüthrich, 2022; Tsoeunyane et al., 2022). In electroless polishing, instead of externally applying current or voltage, an oxidizing or reducing agent is introduced into the electrolyte to promote the movement of electrons (e^−^) or holes (h^+^), thereby inducing oxidation (polishing) reactions (Acquesta and Monetta, 2023; Han and Fang, 2019; Tsoeunyane et al., 2022).

$$Oxidation:{Ti}^{0}\to {Ti}^{4+}+{4e}^{-}$$ (2-1)

$$Reduction:Oxidizing\;agent+{ne}^{-}\;\to \;reduced\;agent$$ (2-2)

When a specimen is introduced, it dissociates into metal ions and electrons through a chemical reaction similar to that in conventional chemical polishing (Kinast et al., 2015). However, the added oxidizing/reducing agent in solution absorbs the separated e^−^/h^+^ and creates a flow similar to the electron movement, helping the polishing reaction to continue (Equations 2-1 and 2-2). In the case of ECP, the process occurs within an electrochemical cell, where an electrical potential is applied to the workpiece acting as the anode (Chaghazardi and Wüthrich, 2022; Han and Fang, 2019; Wu et al., 2019; Xu et al., 2024). Under this anodic bias, the metal is oxidized and dissociates into metal ions and electrons. The released electrons travel through the external circuit toward the cathode, where they are consumed in a reduction reaction, as represented in Equations 3-1 and 3-2.

$$Oxidation\;at\;anode:{Ti}^{0}\to {Ti}^{4+}+{4e}^{-}$$ (3-1)

$$Reduction\;at\;cathode:2H^{+}+2e^{-}\to H_{2}$$ (3-2)

Similar to chemical polishing, electrochemical reactions occur over all specimen surfaces exposed to the electrolyte. However, the application of an electrical potential increases the thermodynamic driving force for anodic dissolution and accelerates the reaction rate. The resulting current distribution promotes preferential dissolution at surface asperities, leading to the attenuation of surface topography and a reduction in surface roughness. At the same time, the suppression of crystallographic and grain-dependent dissolution contrast improves surface gloss and reflectivity, while spatially uniform anodic dissolution enhances overall surface uniformity. Furthermore, the extent of polishing can be controlled by regulating the total electric charge passed. Accordingly, a “well-polished” surface is generally characterized by reduced roughness, increased gloss or reflectivity, and improved surface uniformity, as illustrated in Figure 2b (Han and Fang, 2019; Landolt, 1987; Lebedeva et al., 2021; Lochyński et al., 2019).

### ECP mechanism and main parameters

**FIGURE 3:**
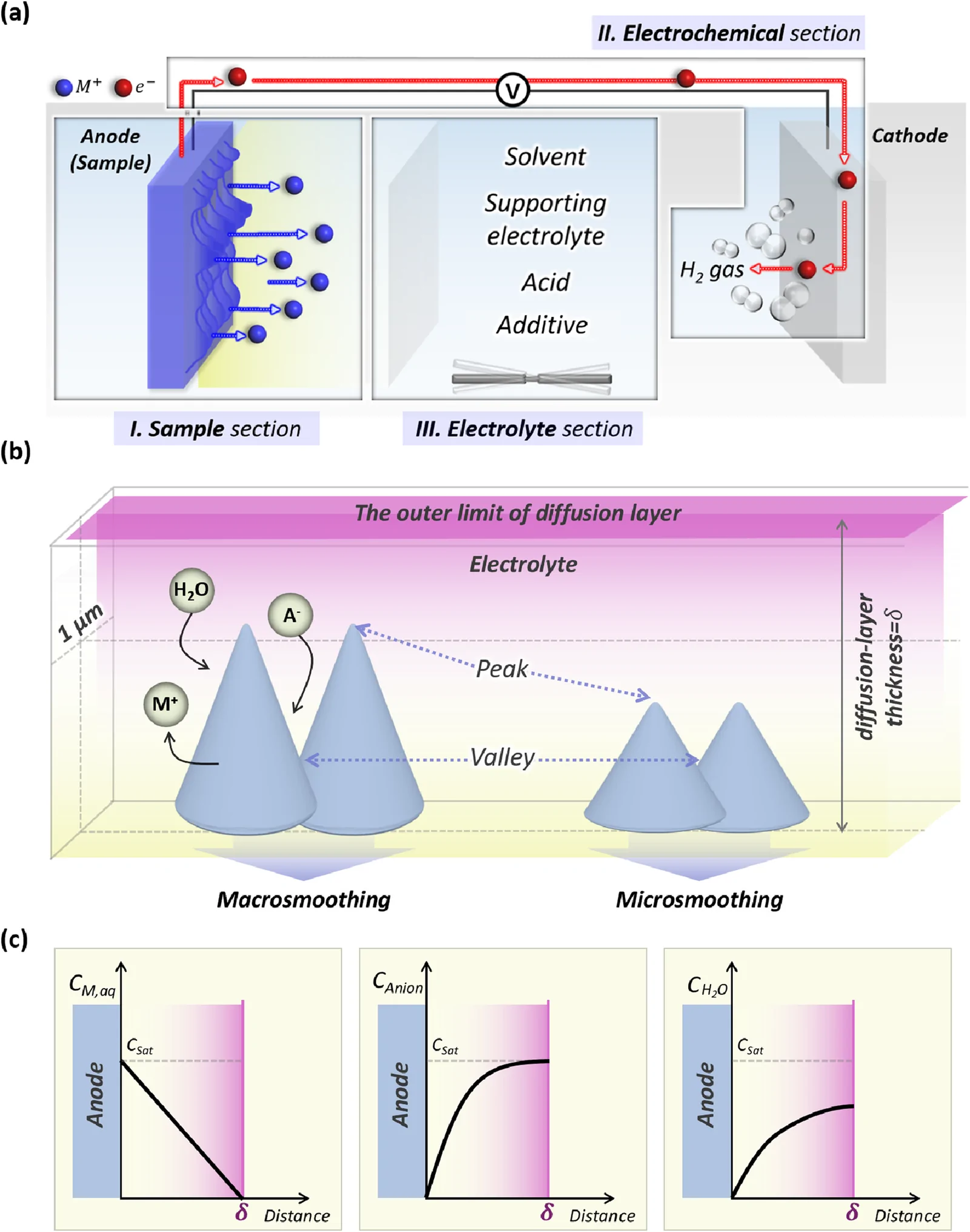
**(a)** Schematic illustration of the electrochemical polishing (ECP) cell; **(b)** classification of surface smoothing at the macro- and micro-scales according to the characteristic dimensions of the surface irregularities relative to the diffusion-layer thickness, δ. **(c)** Schematic concentration profiles representing possible mass-transfer-limiting mechanisms during ECP, including the transport of anodically generated metal ions or dissolution products away from the anode and the supply of acceptor anions or water toward the anode surface. $M_{\text{aq}}$ , anodically generated metal ion; $A^{-}$ , acceptor anion; $H_{2}O$ , water acting as a limiting species; $MA_{y}$ , metal–anion complex and $C_{\text{sat}}$ , saturation concentration.

Because electrochemical reactions involve the interplay of both chemical and electrical processes within the cell, it is essential to consider how various process parameters influence surface quality. As illustrated in Figure 3a, the ECP process is carried out by immersing the workpiece in an electrolytic bath. The workpiece serves as the anode (i.e., working electrode), while the counter electrode completes the circuit and functions as the cathode. The process proceeds through the application of a constant or pulsed electrical potential/current (AC/DC, pulse, or step mode). Upon application of the potential/current, the Ti undergoes anodic dissolution, forming Ti^4+^ ions that subsequently interact with the electrolyte, resulting in the polishing effect. The released electrons flow through the external circuit toward the cathode, where they participate in reduction reactions, typically leading to hydrogen evolution (Equation 3). Given that this overall behavior is governed by the coupled chemical and electrical environment, no single set of parameters can be universally applied under all conditions. The framework adopted here therefore groups ECP parameters into three families—specimen, electrochemical parameter, and electrolyte—and treats each not as an independent variable to be tuned in isolation, but as one of several routes by which a single interfacial state is controlled. This grouping is not arbitrary; it reflects the main factors that control current distribution and mass transport. Specimen geometry and prior topography influence current distribution and geometry-driven leveling.

Electrochemical parameter like applied potential and waveform determine the operating region of the J–V curve, stirring condition, temperature and others. The electrolyte governs the thickness and stability of the diffusion boundary layer, δ, whose magnitude relative to the surface profile separates the two smoothing regimes. Taken together, the three families collectively determine the interfacial conditions governing current distribution, mass transport, and surface leveling, and the sections that follow examine each in turn.

### Specimen

The specimen, which serves as the workpiece during ECP, is connected as the anode in the electrochemical cell. Its initial characteristics—including geometry, surface roughness, crystallinity, and spatial position within the cell—can significantly affect the polishing outcome and must therefore be carefully considered during process design. Prior to a detailed discussion, it is important to distinguish between macro-smoothing and micro-smoothing mechanisms (Figure 3b), which correspond to anodic leveling and surface brightening, respectively (Landolt, 1987).

Surface irregularities consist of elevated regions (“peaks”) and recessed regions (“valleys”), and the vertical distance between them, referred to as the peak-to-valley distance, represents one aspect of surface roughness. To quantitatively characterize surface topography, statistical parameters such as the arithmetic mean roughness (Ra), maximum height roughness (Ry), and ten-point mean roughness (Rz) are commonly employed, each representing different aspects of the surface profile. Ra describes the mean absolute deviation of the filtered surface profile and is therefore useful for evaluating the overall amplitude of surface irregularities. In contrast, Ry and Rz are more sensitive to pronounced peaks, valleys, and localized defects. However, no single roughness parameter can fully describe surface quality, because surfaces with similar Ra values may differ substantially in local defects, spatial morphology, and optical appearance.

For descriptive convenience, macro-smoothing and micro-smoothing are commonly distinguished according to the characteristic length scale of the surface irregularities being removed (Han and Fang, 2019; Landolt, 1987; Mu et al., 2023; Tegart, 1959; Yang et al., 2017). Following the convention widely adopted in the electropolishing literature, this review classifies the attenuation of surface features larger than approximately 1 µm as macro-smoothing, whereas the removal of submicrometer-scale irregularities and the associated formation of a bright surface are classified as micro-smoothing.

This length-scale dependence follows from Wagner’s analysis of an ideal diffusion-controlled electropolishing process (Wagner, 1954). For a sinusoidal profile of wavelength a and amplitude b , the amplitude decreases with the recession u of the mean surface plane as

$$b=b_{0}\exp\left(-\frac{2\pi u}{a}\right)\;\left(b\ll a,a\ll \delta \right)$$ (4)

where b_0_ is the initial amplitude and δ is the effective boundary-layer thickness. Because the decay coefficient is inversely proportional to a, short-wavelength features are attenuated much faster than long-wavelength features for the same amount of metal removed. Thus, microroughness is removed more rapidly than macroroughness, and a multiscale profile progressively loses its shorter-wavelength components. The condition a≪δ also shows that leveling depends on the relation between surface length scale and boundary-layer thickness. As a approaches δ, the leveling rate becomes smaller than predicted by Equation 4, and in the limiting case a≫ δ, diffusion-controlled leveling becomes ineffective. Accordingly, the distinction between micro- and macro-smoothing should be regarded as a practical length-scale classification rather than a fixed material threshold.

Macro-smoothing is associated with spatial variations in the potential or concentration gradients generated by macroscopic surface geometry and proceeds through differences in local current density and dissolution rate between protrusions and recesses. During ECP, an electric field is established across the specimen surface, including between peaks and valleys. Because of geometric variations, the electric field becomes concentrated at the peaks, resulting in enhanced local anodic dissolution and the subsequent removal of material. When the surface roughness is high, the curvature of the peaks becomes more pronounced, further intensifying charge localization and increasing the difference in dissolution rate between peaks and valleys. This effect promotes preferential dissolution at the peak regions, thereby enhancing surface leveling. In contrast, micro-smoothing, or anodic brightening, is associated with a reduction in the influence of microstructural heterogeneities—such as crystallographic orientation, grain boundaries, and phase distribution—on the local dissolution rate, thereby suppressing fine surface relief and microstructural contrast. However, as mentioned earlier, leveling and brightening should not be distinguished solely on the basis of surface roughness, because there is no simple relationship between the profile height measured using a mechanical instrument and the brightness determined from specular reflectance measurements (Landolt, 1987). In practice, as demonstrated in the study by Sard et al., brightness can be affected by whether crystallographic faceting develops, which is governed by the surface profile geometry and the distribution of local surface slopes (Sard et al., 1975).

Although macro-smoothing and micro-smoothing are explained by fundamentally different mechanisms, involving current distribution and surface kinetics or passivation behavior, respectively, they do not necessarily occur as strictly separated sequential stages and may act simultaneously during electropolishing (Landolt, 1987; Sautebin et al., 1980; Sautebin and Landolt, 1982). As the process proceeds, the surface geometry, current distribution, electrode kinetics, and interfacial mass-transfer conditions continuously change, and the relative contribution of each smoothing mechanism therefore also evolves. Accordingly, the interpretation of such complex smoothing behavior requires the analysis of current distribution over the electrode surface using numerical methods such as the finite difference method (Riggs et al., 1981), finite element method (FEM), and boundary element method (Clerc et al., 1984; Clerc and Landolt, 1987; Deconinck et al., 1985; Landolt and Clerc, 1985; Sautebin et al., 1980; Sautebin and Landolt, 1982).

Current distribution can be classified as primary, secondary, or tertiary depending on the variables considered in the electrochemical system. Primary current distribution accounts for cell geometry and ohmic conduction through the electrolyte. Secondary current distribution additionally incorporates activation overpotential and electrode kinetics described by relationships such as the Butler–Volmer or Tafel equations. Tertiary current distribution further considers the coupling between charge-transfer kinetics and mass transport. In the secondary current distribution, both electrolyte resistance and kinetic polarization resistance determine the distribution of current, and their relative importance can be evaluated using the Wa (Equation 5) (Landolt, 1987; Wagner, 1951). When Wa ≪ 1, ohmic and geometric effects are dominant, and the current distribution approaches the primary current distribution, making current concentration at protruding regions more pronounced. In contrast, when Wa ≫ 1, kinetic polarization resistance becomes dominant, suppressing local increases in current density and producing a more uniform current distribution. Therefore, at a high Wa, the macro-smoothing contribution arising from geometry-induced current concentration may decrease.

$$Wa=\frac{Kinetic\;polarization\;effect}{Ohmic/geometrical\;effect}=\frac{\kappa }{L}\left(\frac{d\eta }{dj}\right)=\frac{1}{\rho_{e}\varepsilon_{0}}\left(\frac{d\eta }{di}\right)$$ (5)

$$\kappa :\text{electrolyte conductivity},L:\text{characteristic length},\frac{d\eta }{dj}:polarization\;resistance$$

$$\rho_{e}:\text{electrolyte resistivity},\varepsilon_{0}:\text{initial profile of height},\frac{d\eta }{di}:\text{slope of the current}-\text{voltage curve}$$

Wa≪1,Ohmic,geometrical effect dependent

Wa≫1,Kinetic polarization dependent

During the initial leveling of highly curved or rough surfaces, macro-smoothing behavior can be interpreted on the basis of the primary or secondary current distribution and the Wa when concentration polarization and mass-transfer effects are relatively insignificant and the local current density is governed primarily by surface geometry and electrode kinetics. Under these conditions, geometry-induced current concentration and the resulting difference in local dissolution rate between protrusions and recesses dominate macroscopic surface leveling. However, as electropolishing proceeds, the accumulation of dissolution products or depletion of reactant species at the electrode–electrolyte interface generates concentration gradients, causing mass-transfer limitations to influence the local current density and dissolution rate. Under these conditions, macro-smoothing must be analyzed using a tertiary current-distribution model that accounts for both charge-transfer kinetics and mass transport, and the leveling rate is governed not only by the height and wavelength of the surface profile but also by the thickness of the mass-transfer boundary layer.

As the current increases and the removal rate of interfacially generated dissolution species or the supply rate of reactant species reaches a mass-transfer limit, electrode dissolution becomes governed by mass transport rather than by charge-transfer kinetics, resulting in the appearance of a limiting-current plateau. The rate-determining species varies depending on the electrolyte system. The limiting step may involve the transport of Ti ions or other dissolution products toward the bulk electrolyte or the supply of acceptor species toward the metal surface. The relevant mechanisms associated with the effects of metal ions, acceptor species, and H_2_O are illustrated in Figure 3c. Such mass-transfer-controlled dissolution reduces the relative influence of crystallographic orientation and surface defects on local reaction-rate differences and is therefore favorable for micro-smoothing or anodic brightening. This behavior has traditionally been explained primarily by the salt-film mechanism and the acceptor mechanism—the latter being the basis on which Equation 4 was derived (Hoar and Mowat, 1950; Landolt, 1987; Piotrowski et al., 1998; Wagner, 1954).

Within this framework, the effects of initial surface condition reported in the following studies can be interpreted not simply in terms of $R_{a}$ , but in terms of how the geometry and characteristic scale of the surface profile influence current distribution and mass-transfer-controlled leveling. Urlea and Brailovski electropolished AM Ti-6Al-4V specimens containing surfaces built at 0°, 45°, 90°, and 135° in a V-shaped anode–cathode configuration (Urlea and Brailovski, 2017). Build orientation produced markedly different as-built surface conditions and roughness levels, with the roughest surfaces showing the largest fractional roughness reduction, of approximately 92%. This result should not, however, be interpreted as evidence that $R_{a}$ itself acts as an independent polishing variable; rather, $R_{a}$ reflects the underlying profile geometry established by the build orientation. Within the present framework, the different leveling responses may be understood as arising from differences in surface geometry and their resulting effects on local current distribution and mass transport. Build orientation is therefore treated here as a manufacturing variable that establishes the initial topography and thereby influences the subsequent leveling response.

Lee and Shin electropolished Nitinol specimens with initial roughness values of 1 and 2 µm for 300 s (Lee and Shin, 2011). The initially rougher specimen decreased gradually to approximately 0.98 µm, whereas the initially smoother specimen showed a rapid reduction during the first ∼50 s followed by only limited further improvement. The two specimens did not converge to the same final roughness, but both showed diminishing improvement with time. Qualitatively, this behavior is consistent with a declining leveling rate as the dominant asperities are progressively removed. The observed plateaus indicate that extending the polishing time alone becomes increasingly ineffective under the selected conditions and that further improvement requires modification of the interfacial, hydrodynamic, or electrical conditions.

Wang et al. investigated the effect of inter-electrode distance on the ECP of Ti-6Al-4V specimens in an acid–alcohol electrolyte, and presented the results using three-dimensional surface micro-profiles and roughness analyses (Wang et al., 2021). The distance between the anode and cathode was varied from 2 to 5 cm, while other parameters, including applied voltage (20 V), temperature (20 °C), and polishing time (4 min) were kept constant. When the inter-electrode distance was 2 cm, surface defects such as bubble-induced pits, bumps, and over-polishing were observed because of oxygen evolution at the anode and localized temperature increases within the narrow inter-electrode gap. As the distance increased to 3–4 cm, pitting decreased and the polishing quality improved. However, when the distance was kept at 5 cm, the electric field distribution became more uniform, resulting in reduced current density and weakened polishing reactions. Consequently, this led to incomplete polishing and the formation of surface defects. These results indicate that determining the optimal inter-electrode distance is critical in the design of an ECP process, as it directly influences current density and energy distribution, which in turn govern surface quality.

The specimen establishes the initial conditions of the current-distribution problem. Its asperity wavelength controls the decay rate predicted by Equation 4, while its magnitude relative to δ determines the validity of that relation. Surface curvature affects geometry-driven current concentration, while the Wagner number ((Wa)) determines the relative importance of electrode kinetics and ohmic effects in the resulting current distribution. However, specimen geometry alone cannot ensure uniform micro-smoothing and brightening unless an appropriate transport-controlled polishing regime is established.

### Electrochemical parameters

**FIGURE 4:**
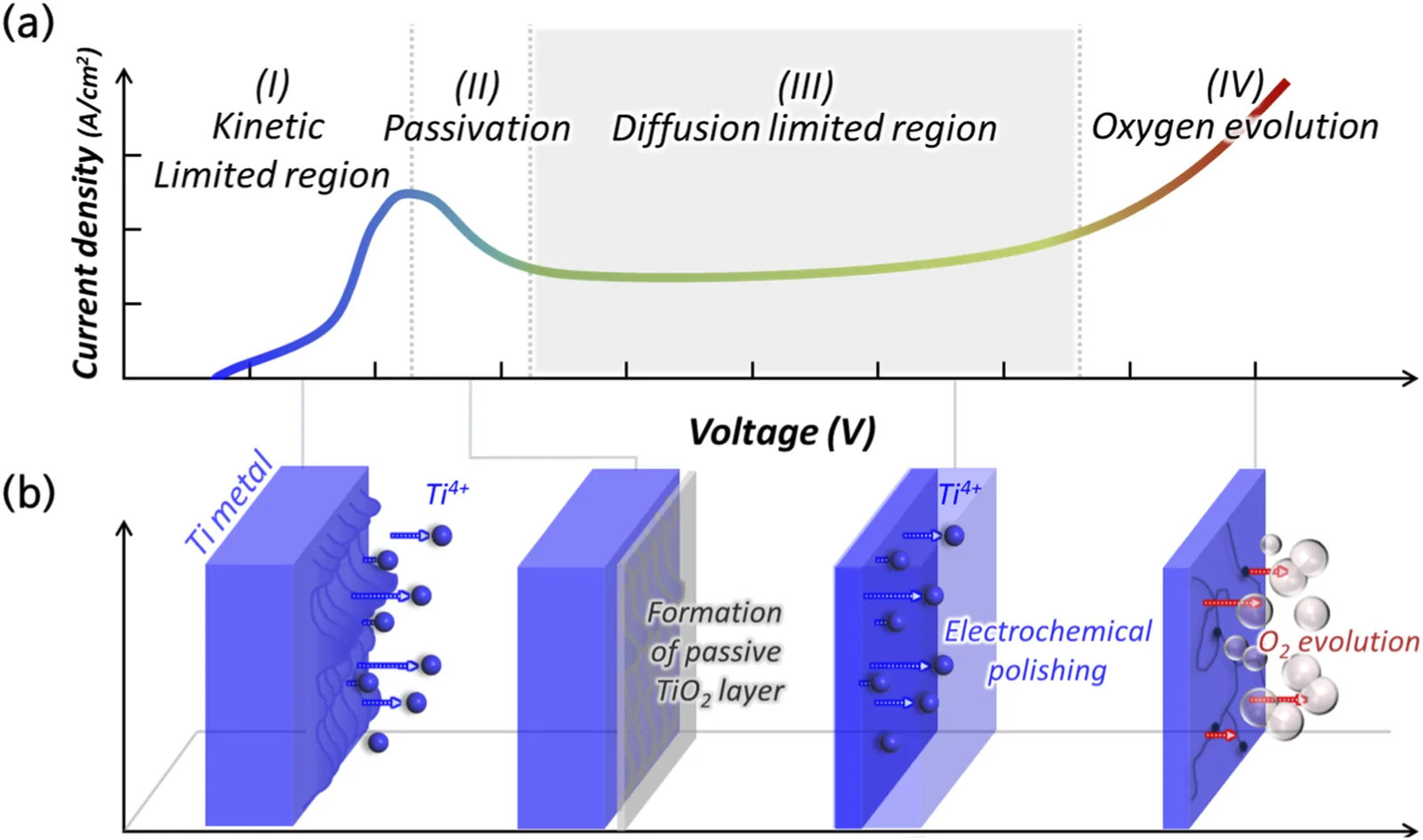
**(a)** Current–voltage (J–V) curve for the electrochemical dissolution of Ti and its alloys; **(b)** corresponding electrochemical reactions occurring on the metal surface.

ECP is initiated by applying a positive potential to the anode thereby driving anodic oxidation reactions. However, in addition to the desired anodic dissolution associated with ECP, competing processes such as passivation, gas evolution, and surface degradation may also occur. Therefore, it is essential to distinguish among these reactions and selectively promote the targeted ECP mechanism. To understand and control these phenomena electrochemically, Jacquet and subsequent researchers analyzed current density–potential (J–V) curves in electrochemical cells during the early stages of process development (Acquesta and Monetta, 2021; 2023; Cuthbertson and Turner, 1966; Han and Fang, 2019; Yang et al., 2017). These analyses enabled the identification of the voltage ranges, corresponding current densities, and underlying mechanisms governing metal dissolution, which were subsequently applied to optimize polishing conditions. Figure 4a,b presents representative J–V curves typically observed during the ECP of Ti. The curves can be broadly divided into four regions: the kinetic-limited region (I), passivation region (II), diffusion-limited region (III), and oxygen evolution region (IV). The kinetic-limited region (I) is characterized by a rapid increase in current density with increasing applied potential, leading to active anodic dissolution and surface etching. In this regime, Ti readily dissolves into the electrolyte, and localized pitting may occur due to surface irregularities introduced during prior AM process. In the passivation region (II), the current density decreases or stabilizes with increasing potential due to the formation of a protective oxide layer on the Ti surface. The diffusion-limited region (III), commonly referred to as the limiting current plateau, corresponds to the effective ECP window. In this region, the current density remains nearly constant despite increasing applied potential, as mass transport across the metal–electrolyte interface becomes the rate-limiting step (Piotrowski et al., 1998). Under these conditions, two interfacial layers with distinct physical characteristics should be distinguished. The compact anodic oxide film is a solid barrier formed directly on the metal surface and primarily governs the passivation behavior of region (II). In contrast, the viscous interfacial layer is a liquid-phase region adjacent to the surface, in which dissolution products accumulate and may precipitate as a salt film upon saturation, or acceptor species become depleted, as described by the salt-film and acceptor mechanisms discussed above. In region (III), mass transport across this liquid-phase layer becomes rate-limiting, thereby enabling preferential dissolution of surface asperities and controlled surface leveling. At higher potentials, the oxygen evolution region (IV) is reached, where the applied potential becomes sufficient to drive the anodic oxidation of water (or other solvent species) in parallel with metal dissolution, so that oxygen evolution proceeds concurrently at the anode. The oxygen bubbles generated tend to adhere to the surface, promoting localized pitting and increasing the likelihood of electrolyte decomposition. In regions (I) and (IV), the combined effects of ion migration and diffusion of dissolved Ti species lead to non-uniform dissolution and surface damage (Karim et al., 2020). In contrast, in region (III), Ti cations are uniformly dissolved under mass-transport-controlled conditions into the bulk electrolyte through the interfacial viscous layer, resulting in stable and controlled polishing. Region (III) is necessary but not sufficient, however. Piotrowski et al. polished Ti at 2, 6, and 8 V under limiting-current conditions in a 3 M H_2_SO_4_–methanol electrolyte. At 2 V, the surface exhibited pits and partial dark-film coverage; at 6 V, it became brighter with smaller pits but retained some waviness; and only at 8 V was a bright and smooth finish obtained. Thus, identifying the limiting-current plateau defines a candidate regime for electropolishing, whereas the applied potential within that regime still strongly influences whether effective leveling is achieved (Piotrowski et al., 1999). Region (III) is the operating point at which the mechanisms of the preceding section become available at all. Outside it, the surface is either etched (I), passivated (II), or pitted by evolving oxygen (IV), and no choice of specimen geometry or electrolyte composition will produce leveling. The parameters that follow—waveform, agitation, temperature, and the electrolyte itself—are best understood as the means of reaching this plateau and holding the process within it.

Taylor et al. applied pulse and pulse-reverse waveforms during the ECP to investigate the influence of on- and off-times, duty cycle, and average current density on the anodic interfacial diffusion layer (Taylor et al., 2011). Their central observation restates, in process terms, the validity condition of Equation 4. Upon pulse application, dissolved metal ions diffuse out to the electrolyte, forming a concentration-gradient-driven diffusion layer at the interface. The thickness of this diffusion layer relative to the initial surface roughness dictates the polishing outcome. When the diffusion layer thickness exceeds the initial surface roughness–completely enveloping the peak-and-valley profile–the process operates under mass-transport-controlled conditions. In this regime, current density becomes preferentially concentrated at surface asperities, promoting selective dissolution and enhanced surface leveling. Conversely, if the diffusion layer thickness is comparable to or smaller than the initial surface roughness, the diffusion layer conforms to the surface topography, independently enveloping peaks and valleys. This results in uniform dissolution across the surface, yielding minimal roughness reduction. Consequently, precise control of the voltage waveform is essential for achieving leveling. The study also demonstrated that extending pulse duration proportionally increases diffusion layer thickness, directly improving polishing quality. In the language of the framework, the pulse waveform is not an independent variable acting on roughness; it is a means of increasing δ until the condition a ≪ δ is satisfied for the wavelengths that remain on the surface. However, because Ti readily forms a dense, stable passive oxide layer, residual oxide layers often persist on the surface, leading to increased roughness. To mitigate this problem, pulse/pulse-reverse (PPR) waveforms incorporating periodic cathodic pulses have been employed to disrupt the passive film and improve surface smoothness. Acquesta et al. systematically varied the agitation rate (0–1,150 rpm) to investigate the effect of mass transfer during ECP of additively manufactured Ti-6Al-4V dog-bone specimens in ethylene glycol (EG) based electrolyte (Acquesta et al., 2023). At relatively low agitation rates (0–450 rpm), polishing efficiency gradually decreased, whereas the roughest surface morphology was observed at approximately 800 rpm, likely due to unstable interfacial transport and disruption of the viscous interfacial layer. Interestingly, at higher agitation rates (up to 1,150 rpm), polishing performance improved substantially, resulting in the lowest surface roughness. Under optimized conditions, approximately 64% surface roughness reduction was achieved after 60 min of ECP treatment. Agitation affects electropolishing by changing mass transport and the interfacial viscous layer. Stronger agitation usually thins the diffusion layer and speeds up dissolution, but here the result was different: polishing was worst near 800 rpm and improved again at 1,150 rpm. This shows that good agitation must increase mass transport while keeping the interfacial layer stable.

### Electrolyte

The composition and physicochemical properties of the electrolyte play a critical role in determining the polishing performance and surface quality (Han and Fang, 2019). In general, the electrolyte selection depends on the type of metal being processed; however, both single-component and multi-component systems are widely employed by combining organic and inorganic electrolytes (Han and Fang, 2019; Landolt, 1987). Electrolytes can be broadly classified into aqueous and non-aqueous systems. Aqueous electrolytes represent the conventional approach and typically involve strong acids such as perchloric acid, glacial acetic acid, hydrofluoric acid, and sulfuric acid (Benedetti et al., 2017; Dong et al., 2019; Longhitano et al., 2015; Urlea and Brailovski, 2017; Wu et al., 2019; Zhang et al., 2018). However, acid-based systems pose significant health and environmental risks because of their toxicity and corrosivity, thereby necessitating the development of safer alternatives. Furthermore, the viscosity of aqueous electrolytes is often difficult to control, and many such electrolytes are highly corrosive (Yang et al., 2017). Consequently, non-aqueous electrolytes—including DESs, ILs, and EG-based systems—have recently emerged as promising alternatives.

The four functional units in Table 2 should not be regarded as fully independent variables, because their effects converge on the coupled interfacial conditions governing charge transfer, ohmic conduction, mass transport, and film formation. The solvent mainly establishes the baseline viscosity, diffusivity, and hydrodynamic response of the electrolyte and thereby contributes to the effective mass-transfer-layer thickness, δ. The supporting electrolyte primarily controls ionic conductivity and solution-phase ohmic losses and therefore contributes, together with electrode kinetics and geometry, to the Wa in Equation 5. Acidic or otherwise reactive components participate in metal dissolution and may influence the formation, dissolution, or renewal of oxide, salt-rich, or reactant-depleted interfacial layers, depending on the electrolyte system. Additives may further modify polarity, viscosity, conductivity, complexation, adsorption, and film stability. Thus, an ECP electrolyte composition can be interpreted as a specification of a coupled interfacial state rather than merely as an empirical recipe. The studies discussed below are therefore organized according to the functional component that they primarily investigate, while recognizing that changing one component may simultaneously alter several interfacial properties.

Organic electrolyte systems are generally composed of a base solvent, acidic or reactive supporting components and additional additives. Organic solvents such as EG, ethanol, and methanol serve as the base medium, while other components function as additives tailored to achieve specific properties, including corrosion resistance, brightness, reflectivity, hardness, mechanical strength, internal stress control, and wear resistance (Kao and Hocheng, 2003; Lee and Lai, 2003). The base solvent constitutes the primary component of the electrolyte and plays a crucial role in governing interfacial behavior. The use of organic solvents suppresses excessive corrosion and uncontrolled oxide formation while enabling higher viscosity compared to aqueous systems. Among the various parameters, viscosity is particularly important in ECP processes. As discussed previously, ECP relies on the potential difference between peak and valley regions of the surface. This phenomenon is influenced not only by the applied electric field but also by the viscosity of the electrolyte, which governs ion mobility and mass transport characteristics (Han and Fang, 2019; Landolt, 1987; Tegart, 1959).

**FIGURE 5:**
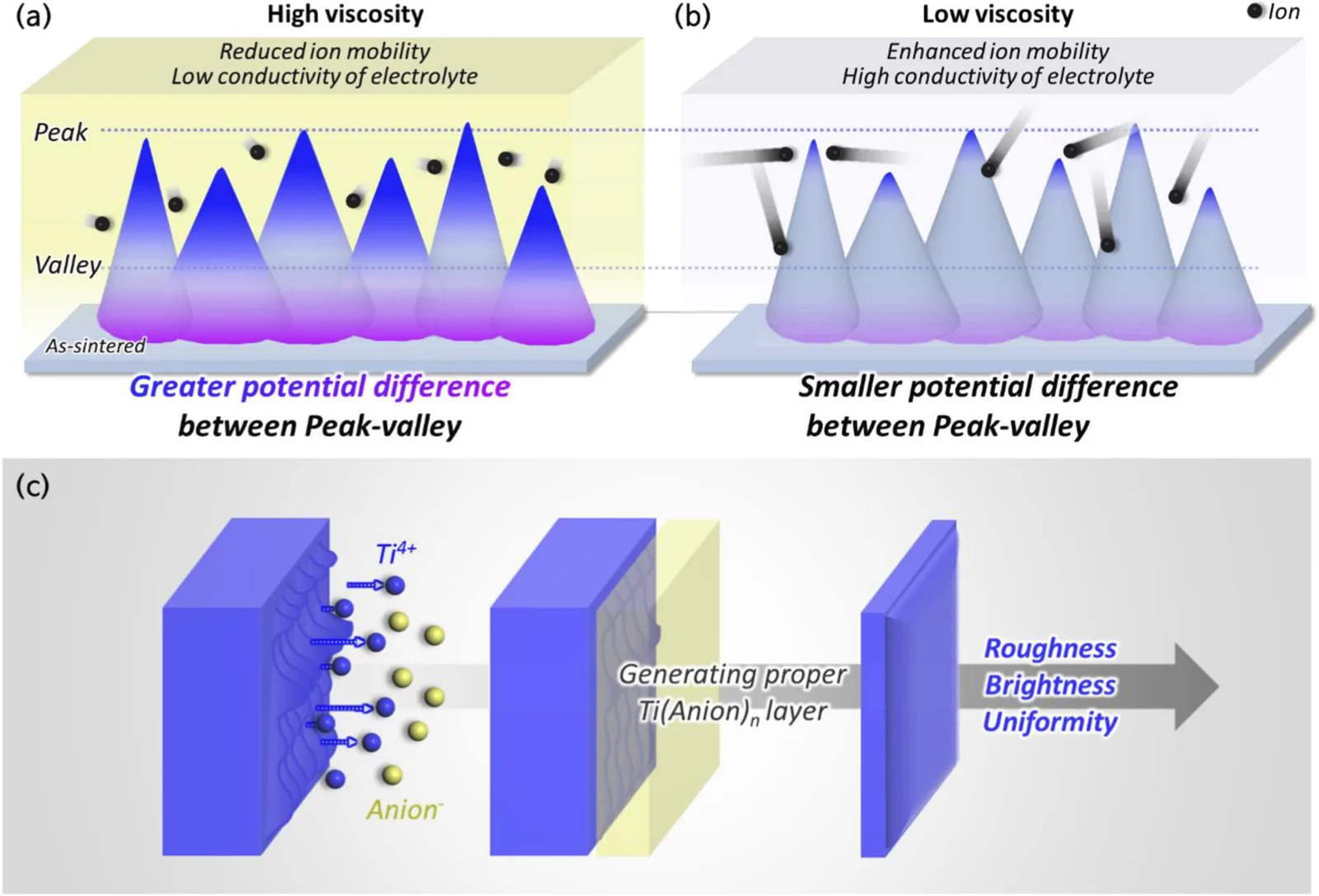
Schematic illustration of electrochemical potential distribution at Ti surface as a function of electrolyte viscosity. Red indicates negative potential, while blue represents positive potential. **(a)** High-viscosity electrolyte; **(b)** low-viscosity electrolyte; **(c)** schematic illustration of electrochemical polishing in additive-containing electrolytes under diffusion-limited conditions.

Figure 5 illustrates the distribution of the electric field as a function of electrolyte viscosity, assuming identical applied potentials in both cases. In high-viscosity electrolytes, ion mobility is restricted, resulting in reduced interfacial conductivity. This enhances the potential difference between peaks and valleys, promoting preferential dissolution of surface asperities and thereby facilitating effective surface leveling (Figure 5a). Conversely, in low-viscosity electrolytes, increased ion mobility leads to higher interfacial conductivity, which diminishes the potential gradient between surface features and reduces the efficiency of selective dissolution (Figure 5b). However, these properties are not constant during electropolishing and may also vary with temperature and operating conditions. Increasing temperature generally decreases viscosity while increasing ionic conductivity and diffusion coefficients. Consequently, mass transport, interfacial-layer characteristics, and current distribution may change even when the nominal electrolyte composition and applied potential remain unchanged. This coupling becomes particularly important during prolonged electropolishing, in which Joule heating and electrochemical reactions can progressively increase the electrolyte temperature. Therefore, temperature should be controlled or at least reported to ensure reproducible process conditions. Within this coupled framework, electrolyte composition remains a key controllable parameter because it can be deliberately adjusted through the addition of salts and other additives. As illustrated in Equations 6-1 and 6-2, the introduction of anions into the electrolyte modifies the ECP behavior of Ti alloys by changing electrical conductivity, pH, polarity, and interfacial-film characteristics. Appropriate control of these properties can promote a brighter, smoother, and more uniform surface finish.

$$Ti\to {Ti}^{4+}+{4e}^{-},at\;anode$$ (6-1)

$${Ti}^{4+}+{anion}^{-}\left(by\;electrolyte\right)\to {Ti\left(anion\right)}_{n},at\;anode$$ (6-2)

NaCl, CaCl_2_, and/or MgCl_2_ are used as supporting electrolytes to minimize ohmic drop. Acidic components directly participate in anodic dissolution reactions, facilitating the conversion of metal into metal ions. Additional components including water, ethanol (C_2_H_5_OH), and methanol (CH_3_OH) are introduced to regulate polarity, solubility, viscosity, and overall reactivity, thereby enabling control over surface gloss, uniformity, and polishing efficiency. Although these electrolyte systems differ widely in chemistry—combining various organic solvents, supporting salts, acids, and functional additives in different proportions—they share a common design objective: to establish a stable diffusion-limited regime corresponding to region (III) in the anodic polarization curve. Reaching and sustaining this plateau is central to ECP optimization, since it dictates the balance between dissolution rate, surface leveling, and finish uniformity (Han and Fang, 2019; Hwang and Kim, 2025; Landolt, 1987; Mu et al., 2023; Yang et al., 2017).

Kim et al. investigated the effect of ethanol, a representative polar solvent, added to EG/NaCl-based electrolytes at concentrations ranging from 0 to 30 vol% for the ECP of Ti sheets (Kim et al., 2015). ethanol was introduced as an additive to modulate electrolyte polarity and promote initial adsorption reactions at the interface. When an optimal concentration (20 vol%) was employed, the overall polarity decreased, and the thickness of the high-viscosity byproduct layer, primarily composed of TiCl_4_, was reduced, enabling nanoscale ECP. However, at higher concentrations (≥30 vol%), an ethanol-rich barrier layer formed, which reduced ion flux and hindered the ECP reaction. Consequently, the addition of 20 vol% ethanol was found to effectively regulate the TiCl_4_ layer formed on the surface, resulting in a mirror-like finish. This result exemplifies the additive function in Table 2: ethanol modifies electrolyte polarity and interfacial adsorption, thereby regulating the formation and transport resistance of the TiCl_4_-rich interfacial layer. Both insufficient and excessive ethanol addition produce interfacial conditions that are less favorable for selective dissolution, accounting for the non-monotonic response and the optimum observed at 20 vol%.

Zhang et al. further examined the role of chloride ions in the ECP of AM Ti-6Al-4V alloys using an EG-based electrolyte (Zhang et al., 2020). Consistent with previous studies, Cl^−^ ions were introduced to enhance conductivity and optimize polishing performance. At excessively high concentrations (≥0.5 mol/L), a thick TiCl_4_ layer formed at the initial stage of the reaction, inhibiting effective polishing and leading to pore formation and surface irregularities. In contrast, at low concentrations (≤0.3 mol/L), the current density decreased, slowing the reaction rate and resulting in non-uniform polishing. Notably, at an optimal concentration of approximately 0.4 mol/L, the TiCl_4_ layer was dynamically formed and removed, enabling the formation of a smooth and uniform surface with a roughness on the order of ∼1 µm. This result illustrates the role of supporting electrolytes in Table 2. The Cl^−^ concentration affects both electrolyte conductivity and the formation of the TiCl_4_-rich surface layer. Therefore, the best polishing condition is not achieved at the highest Cl^−^ concentration, but at an intermediate concentration where the surface layer can form and be removed continuously.

**FIGURE 6:**
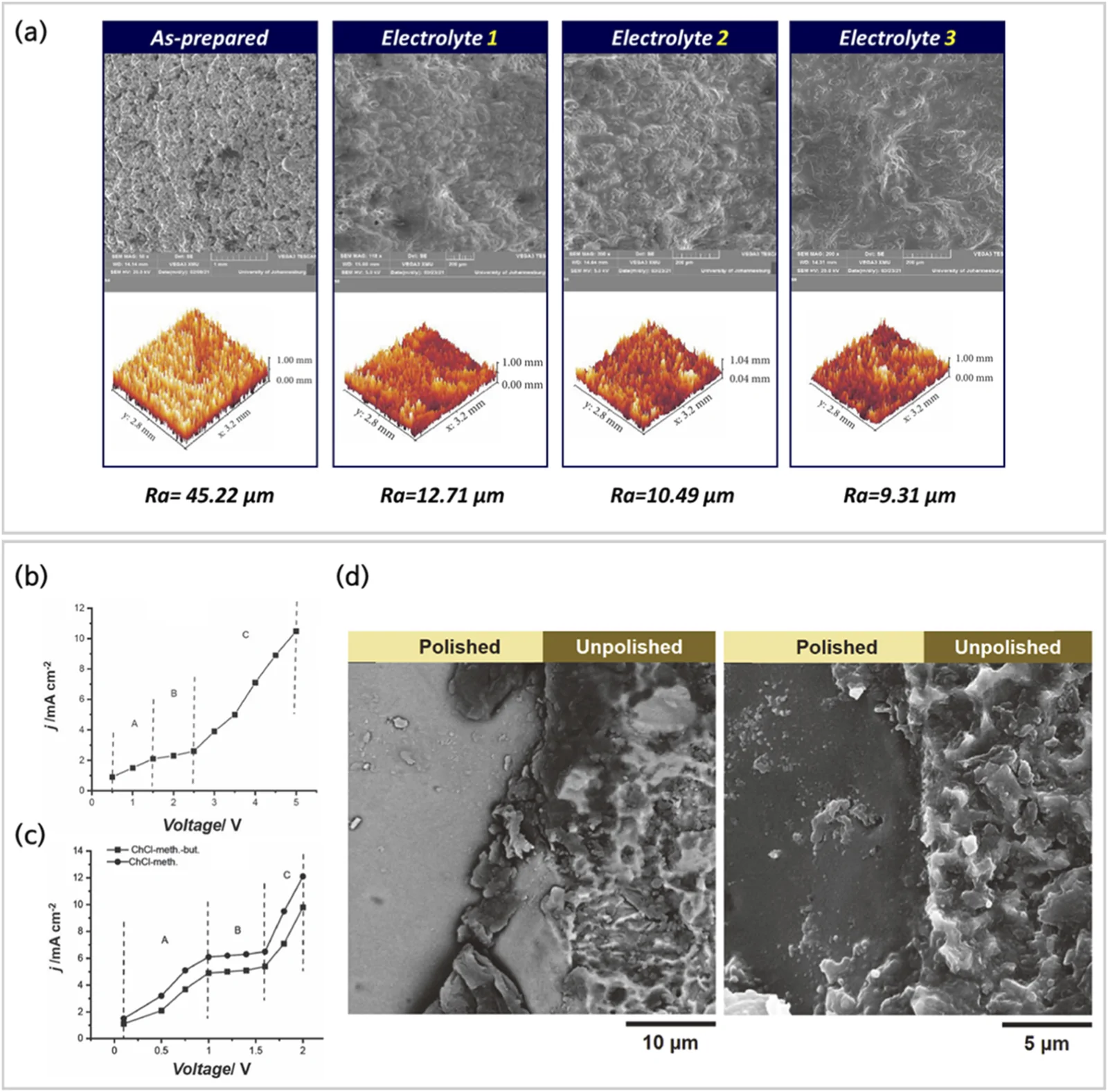
**(a)** SEM and corresponding atomic force microscopy (AFM) images of the Ti surface before and after electropolishing using three different baths (Tsoeunyane et al., 2022). Effects of solvents for V–J curve: ChCl + EtOH **(b)**, ChCl + MeOH + BuOH and ChCl + MeOH **(c)** (Karim et al., 2021). **(d)** SEM image showing the boundary between polished and unpolished regions of pure Ti. **(Left)** ChCl + MeOH + BuOH, at 1.2 V **(Right)** ChCl + EtOH at 2 V.

EG-based chemistry has also been extended to deep eutectic and ionic-liquid systems. For example, a deep eutectic solvent composed of choline chloride (ChCl) and EG at a molar ratio of 1:2 provides a relatively mild and environmentally benign polishing environment (Karim et al., 2020). Through polarization curve analysis, optimal processing conditions were identified. As a result, the average surface roughness (Ra) was significantly reduced from an initial value of 118.8 nm–5.7 nm, demonstrating highly effective surface smoothing. Tsoeunyane et al. conducted ECP using a triethanolamine-ethanol electrolyte to reduce the roughness of AM Ti-6Al-4V material and reported that adding salt resulted in a five-fold reduction in average roughness and the formation of an oxide film that protects the surface (Table 4; Figure 6) (Tsoeunyane et al., 2022). In this study, they proposed an electrolyte mixture of various salts, acid and organic solvents. The electrolyte was composed of different acids, salts such as perchloric acid, NaF, and NH_4_F by adjusting the ratio for ion regulation (Figure 6a). The applied current density, operating temperature and time were kept constant. Basically, an organic solvent (triethanolamine) with low moisture content was used to control excessive growth of the oxide film, and its high viscosity enabled uniform polishing within the ECP cell. In electrolyte 1, Cl^−^ and F^−^ anionic species from the salts strongly reacted with Ti^4+^ ions resulting in the formation of TiCl_4_ and TiF_4_ which could passivate the Ti surface. In addition, the pit-like defects were observed on the surface of the specimen after the ECP which would be due to the interruption of the efficient Ti dissolution. Accordingly, electrolytes 2 and 3, which did not contain perchloric acid, avoided pit formation and showed comparable or slightly lower surface roughness compared with electrolyte 1. Among them, electrolyte 3 exhibited the lowest Ra value of 9.31 µm, corresponding to approximately an 80% reduction compared with the as-built specimen (Ra = 45.22 µm). This study illustrates the combined effect of acid and fluoride salts on the polishing reaction. Because the perchloric acid content and fluoride concentration were varied simultaneously, the improved surface quality cannot be attributed to a single component. Instead, the results indicate that the balance among perchloric acid, NaF, and NH_4_F strongly affects surface dissolution, film formation, and pit development.

**TABLE 4:** Electropolishing parameters (Tsoeunyane et al., 2022 **)**.

Parameters	Electrolyte 1	Electrolyte 2	Electrolyte 3
Triethanolamine	180 mL	180 mL	180 mL
Perchloric acid	10 mL	-	-
Sodium fluoride (NaF)	1.17%	1.17%	2%
Ammonium fluoride (NH_4_F)	5%	10%	10%
Oxalic acid	16%	16%	16%
Ethanol	15 mL	15 mL	15 mL
Current density	0.2 A cm^-2^	0.2 A cm^-2^	0.2 A cm^-2^
Time	30 min	30 min	30 min
Temperature	30.0 °C ± 2 °C	30.0 °C ± 2 °C	30.0 °C ± 2 °C

Hwang et al. utilized choline chloride and EG-based DES electrolytes and examined surface changes according to voltage, processing time, electrolyte ratio, added distilled water concentration, and temperature (Hwang and Kim, 2025). An interesting point is that distilled water added to organic electrolytes plays an important role. Distilled water, as a polar molecule, weakens the hydrogen bonds between choline chloride and EG, appropriately reducing the electrolyte’s viscosity and stabilizing the dissolution reaction. Therefore, they reported that increasing the distilled water content shortens the time required to reach steady state, with approximately 30% identified as the optimal addition. Across these studies, each reports an optimum—20 vol% ethanol, ∼0.4 mol/L chloride, 30% water—yet almost always infers the interfacial layer from composition or outcome rather than measuring it directly. The result is many locally optimized recipes but few rules for predicting an optimum in an untested electrolyte—a gap that will close only when interfacial-layer thickness and stability are treated as measured quantities.

ILs and DESs have recently emerged as promising alternatives for environmentally benign ECP of Ti and its alloys. Karim and co-workers examined this family systematically, holding ChCl fixed as the supporting electrolyte while varying the hydrogen-bond donor across EG, ethanol, methanol–butanol, and propylene glycol. The series therefore functions as a probe of the solvent column of Table 2. Karim et al. investigated the electropolishing behavior of pure Ti using ChCl–ethanol (molar ratio 1:4) and ChCl–methanol–butanol (molar ratio 1:6:2) electrolytes to develop a low-cost and environmentally friendly polishing process (Karim et al., 2021). These organic solvent systems consist of complex ionic species and exhibit wide electrochemical stability windows, making them attractive for Ti ECP applications. The study systematically evaluated the influence of electrolyte composition on polishing behavior by analyzing current density–voltage (J–V) curves for each electrolyte. The polarization behavior was categorized into three regions: metal dissolution (A), electropolishing (B), and electrolyte decomposition (C) (Figure 6b–d). Compared with conventional electrolytes, both systems enabled stable ECP at relatively lower applied potentials. Upon entering the limiting-current plateau region, mass transport across the interface became dominant, enabling the process to reach a stable polishing state. Under optimized conditions, electropolishing was conducted at 2.0 V and 1.2 V for the respective electrolytes. Figure 6d presents scanning electron microscopy (SEM) images before and after polishing. The unpolished surface exhibited distinct grain boundaries and surface irregularities, whereas the polished surface became uniformly smooth and bright without noticeable surface damage or abnormal microstructural features. Surface roughness was significantly reduced from an initial value of 120.5 nm (bare sample) to 0.10 nm in the ChCl–ethanol electrolyte and 0.66 nm in the ChCl–methanol–butanol electrolyte, demonstrating highly effective surface finishing.

In another study, Karim et al. employed a DES electrolyte composed of ChCl and propylene glycol (PG) at a molar ratio of 1:2 for Ti electropolishing (Karim, 2021). This study emphasized the importance of the viscous interfacial layer governing mass transport and selected the DES system due to its relatively wide electrochemical window and favorable environmental characteristics. As a result, surface roughness decreased from an initial value of 455.60 nm–37.92 nm, yielding a mirror-like and highly reflective surface finish. These findings highlight that IL- and DES-based electrolytes provide wide electrochemical windows and stable mass-transport-controlled conditions, making them promising candidates for high-quality and environmentally sustainable Ti ECP processes.

Taken together, these examples suggest that surface quality in Ti-alloy ECP is governed less by individual electrolyte components than by how their combined chemistry regulates the interfacial layer at the anode surface. Optimal polishing typically occurs within a narrow compositional window, in which solvents, salts, acids, and additives collectively stabilize interfacial transport. Deviations from this balance may lead to insufficient surface leveling or transport-limited defects. Building on this perspective, Table 4 summarizes representative ECP conditions reported for Ti and Ti-6Al-4V alloys over the past 2 decades, highlighting the diversity of electrolyte formulations and the corresponding surface finishes achieved.

### Scope and selection of the compiled conditions

The studies in Table 4 were identified through Google Scholar, combining “electropolishing” and “electrochemical polishing” with “titanium” and “Ti-6Al-4V″, and supplemented by the reference lists of the reviews in Table 1. A study was included if it reported ECP of Ti or Ti-6Al-4V, specified the electrolyte composition in enough detail to be resolved into the functional units of Table 2, and reported either a roughness value or an explicit description of the resulting surface. Both additively manufactured and conventionally processed substrates were retained, since one aim of the compilation is to place them in a common frame. The compilation is purposive rather than exhaustive: it seeks to span the range of electrolyte chemistries and operating regimes applied to Ti, not to enumerate every published condition. The resulting 24 studies (2011–2025) are ordered not chronologically but by the functional decomposition of Table 2, so that each entry reads as a choice of solvent, supporting electrolyte, acid, and additive rather than an isolated recipe.

## State-of-the-Art studies on ECP of Ti alloys

### Industrial applications of ECP

**FIGURE 7:**
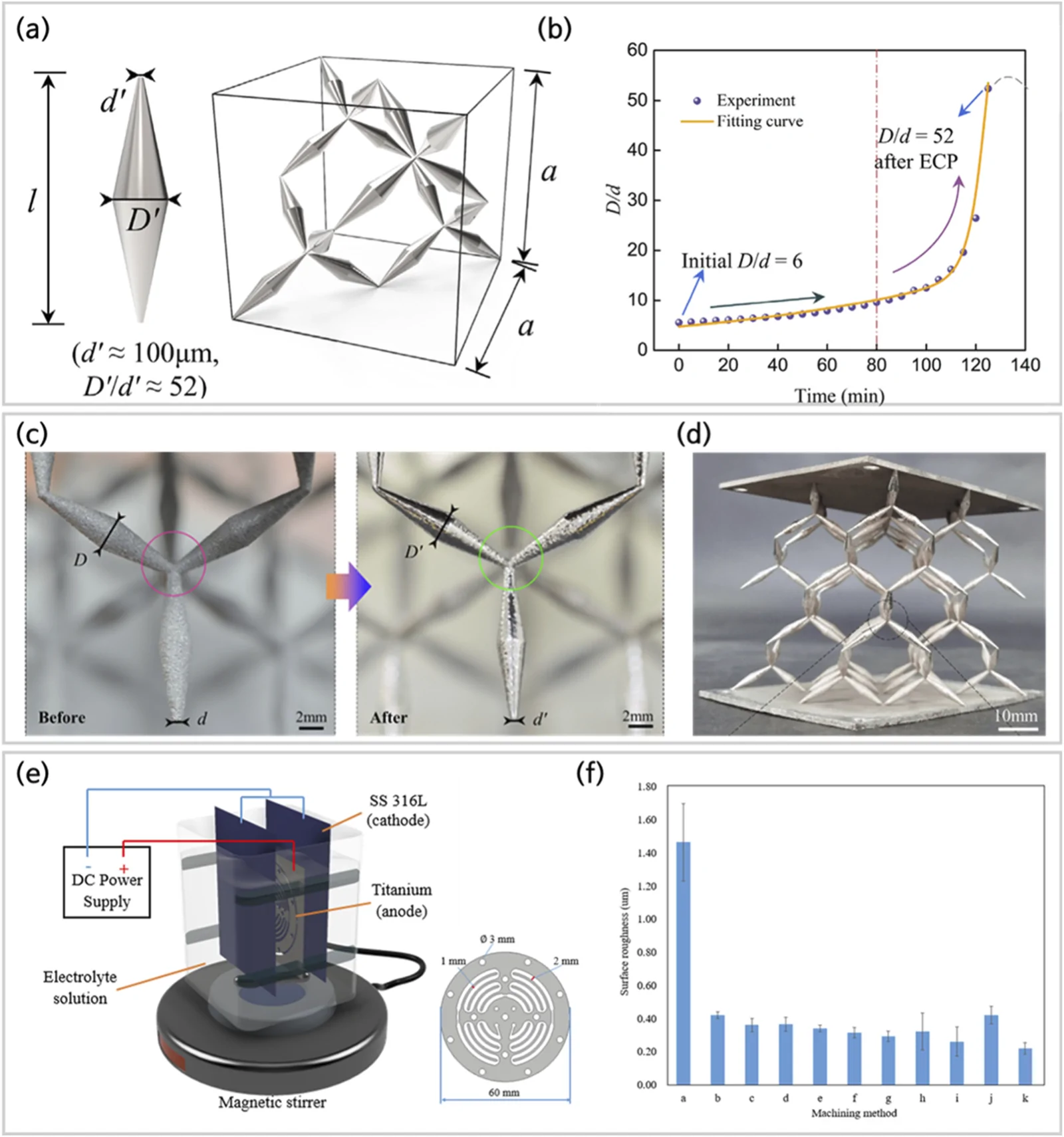
**(a)** Crucial geometric parameters for PMs, and **(b)** D/d ratio variation as a function of etching time. **(c,d)** Optical images of Ti-6Al-4V parts before and after ECP (Jiang et al., 2022) **(e)** Microfluidic channel design for ECP, and **(f)** surface roughness variation of the microfluidic channel sidewall depending on ECP methods and conditions (Mahardika et al., 2021).

ECP has recently been extended from laboratory-scale surface finishing to component-level precision manufacturing for advanced functional materials. Jiang, S., et al. investigated the optimization of ECP parameters for Ti-6Al-4V structures with microscale features, specifically targeting the intersection nodes of pentamode metamaterials (PMs) (Jiang et al., 2022). As illustrated in Figure 7a, PMs exhibit unique functionalities—including seismic isolation, acoustic manipulation, and mechanical cloaking—arising from their architected geometries. However, fabrication of structurally stable nodes below ∼200 µm remains challenging using conventional AM techniques. To address this limitation, ECP was applied using a NaCl–EG electrolyte at 30 °C–40 °C to selectively refine microscale geometries. Following ECP treatment, the D/d ratio increased significantly from 6 to 52, resulting in markedly sharper and more refined structural features (Figure 7b–d). The enhancement became particularly pronounced after approximately 80 min, where the D/d ratio increased nonlinearly. This behavior was attributed to localized electric field concentration at sharp geometrical features, accelerating preferential anodic dissolution at smaller-diameter regions and thereby enhancing structural refinement. Given that currently available AM techniques are generally limited to D/d ratios of approximately 10, the substantial improvement achieved through ECP highlights its strong potential for low-cost precision manufacturing of architected Ti-based materials.

In the biomedical field, ECP has been widely explored to improve the surface quality and biocompatibility of Ti-based microstructures and implants. One study reported the surface refinement of microchannels for biomedical devices using an anhydrous glycol–NaCl electrolyte with the addition of 20 vol% ethanol (Figure 7e,f). (Mahardika et al., 2021) Process parameters including applied voltage (15–25 V), ethanol concentration (15–25 vol%), and machining gap (10–30 mm) were optimized using an orthogonal experimental design. Under optimized conditions (20 V, 20 vol% ethanol, and 10 mm machining gap), surface roughness was reduced by approximately 85%, decreasing from 1.46 µm to 0.22 µm. Furthermore, *in vitro* blood penetration experiments confirmed a significant reduction in contaminant adhesion, suggesting improved surface biocompatibility. Similarly, Tyagi, S et al. employed ECP as a post-processing technique for additively manufactured titanium triply periodic minimal surface (TPMS) structures used in biomedical implants (Tyagi et al., 2025). Gyroid and diamond lattice architectures were evaluated under both graded and non-graded configurations to investigate their mechanical performance. Electropolishing was conducted in a methanol–perchloric acid electrolyte (80:20 vol%) under stirring conditions of 100 rpm. As commonly observed in ECP, the edge effect promoted preferential smoothing of sharp features and surface irregularities, resulting in more uniform geometries. Importantly, no significant changes in particle morphology, phase distribution, or crystallographic structure were observed, indicating that ECP selectively modified surface morphology without altering bulk material properties. Consequently, the process enabled stiffness modulation through selective surface refinement and improved fatigue resistance by mitigating stress concentration sites.

Beyond biomedical and architected materials, ECP has also been applied to structural Ti-alloy components requiring enhanced fatigue performance. L. Yang., et al. optimized the electropolishing conditions for SLM-fabricated Ti-6 Al-4 V components by systematically varying electrode gap (5–10 mm), applied voltage (60–100 V), polishing time (5–20 min), and temperature (27 °C–38 °C) (Yang et al., 2016). Their study highlighted electrolyte flow and thermal management as critical factors governing polishing stability and performance, incorporating both a circulation pump and condenser heat exchanger into the process design. Under optimized conditions (80 V–27 °C and 60 V–38 °C), the fatigue life of Ti-6 Al-4 V specimens increased by more than 300%, demonstrating the effectiveness of ECP in improving structural reliability for industrial-scale components.

Across these applications a common pattern emerges. In each case the electrolyte, waveform, and cell geometry were arrived at by systematic experimental search—orthogonal arrays, parameter sweeps, iterative optimization—and in each case that search was expensive: Ti is a costly substrate, and the number of interacting variables is large. This is the practical cost of the gap identified at the outset. A framework that predicts which region of the process window a given combination will occupy does not eliminate the search, but it can bound it; and where the geometry is complex enough that intuition fails, that bounding is most usefully performed computationally.

### Prediction and optimization of ECP using software

In recent years, ECP research has expanded beyond experimental parameter screening toward predictive process design using computational models. Finite-element platforms such as COMSOL Multiphysics allow the current-distribution and mass-transport frameworks introduced in Section “Specimen” to be applied to geometries that cannot be treated analytically: primary current distribution accounts for electrolyte resistance and cell geometry, secondary distribution adds electrode kinetics, and tertiary distribution further couples concentration gradients and mass transport. Simulation therefore does not constitute a separate theory of electropolishing; it provides a numerical means of applying established theory to realistic process configurations. Its practical credibility can accordingly be assessed by three questions: (i) whether the initial roughness and evolving surface topography are represented realistically; (ii) whether the boundary conditions of as-built AM geometries can be resolved; and (iii) whether the model has been validated sufficiently for its intended role in process optimization.

**FIGURE 8:**
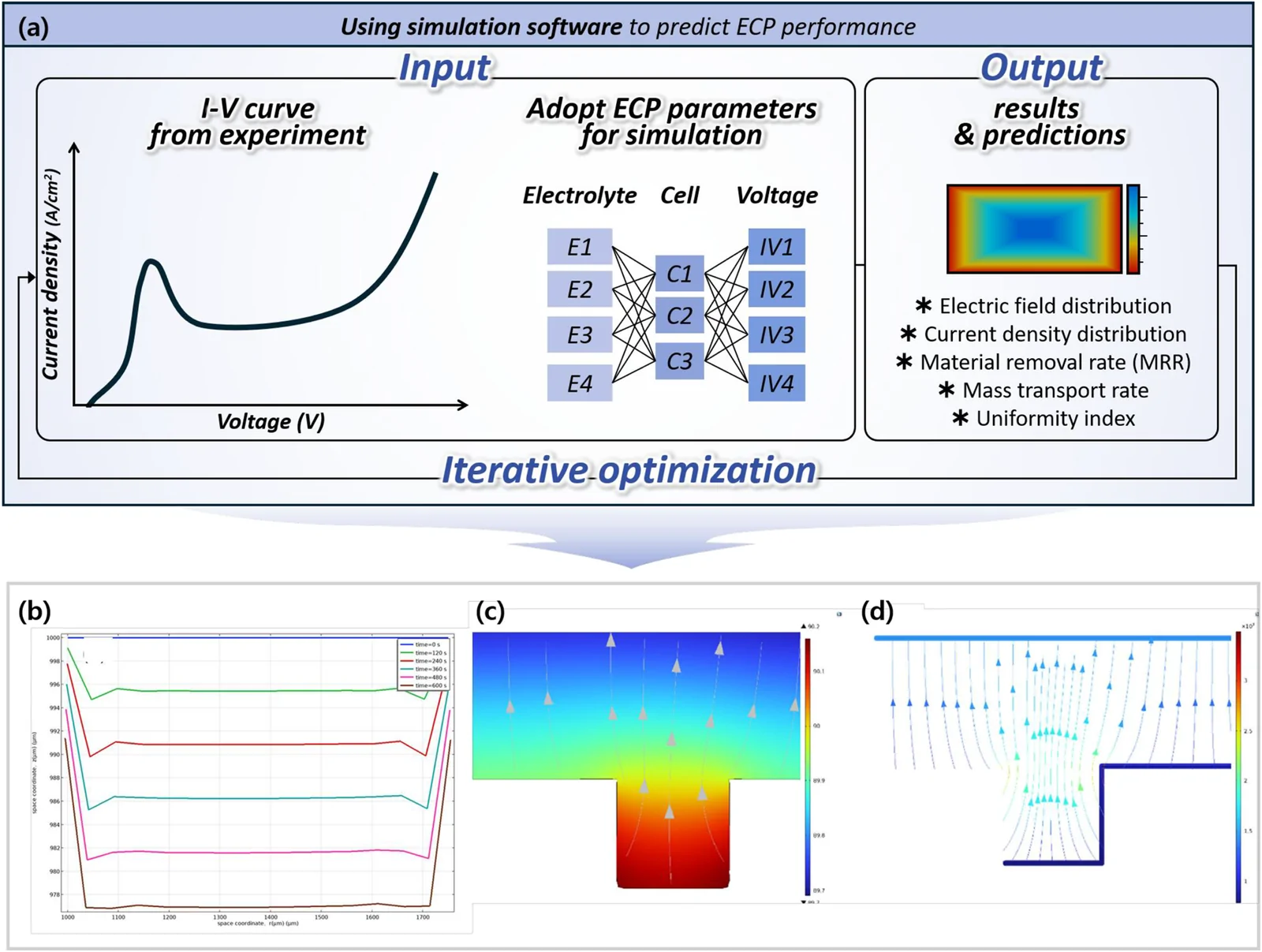
**(a)** Schematic illustration of ECP prediction process using software. Prediction of electrochemical polishing results of Ti–6Al–4V alloy components with micro-grooves structure through COMSOL simulation. **(b)** Material removal rate prediction based on voltage **(c)** Electric field distribution and **(d)** Current density distribution at 90 V (Wang et al., 2024).

Such models provide spatially and temporally resolved quantities that are difficult to measure directly during ECP, including current-density distributions, species concentrations, interfacial-layer thickness, and local material-removal rate (Figure 8). Their principal value is not to eliminate experiments but to reduce the experimental parameter space—particularly relevant for Ti, where repeated trials are costly. Software-based prediction typically follows a systematic workflow (Figure 8a): measured J–V data are used to calibrate the electrochemical kinetic parameters, the geometry and process variables are then defined, and processing conditions are refined through iterative comparison with experiments. Regarding the first question, Tailor et al. calculated the effects of inlet velocity, diffusion coefficient, inter-electrode gap, and initial surface roughness on interfacial-layer formation, predicting thinner layers near the electrolyte inlet and suppression of layer formation at excessive flow velocities (Tailor et al., 2015). This study is notable because the thickness and stability of the interfacial layer were calculated rather than inferred from polishing results, providing a direct numerical connection to the effects of δ, agitation, and electrode geometry discussed in Sections “Specimen” and “Electrolyte”. The predicted layer was, however, based on prescribed transport properties, and its thickness should be regarded as a model-dependent representation rather than a measurement of a unique physical film.

A more demanding test is whether the evolving surface itself is included in the computational domain. Dadi et al. incorporated a measured surface profile from an additively manufactured stainless-steel component directly into the anode boundary of a two-dimensional COMSOL model, using mesh deformation to update the profile according to the calculated local dissolution velocity (Dadi et al., 2023). The simulations reproduced the experimentally observed leveling sequence—higher current density and preferential removal at surface peaks—and the predicted final roughness, reduced by more than an order of magnitude, and dissolved mass agreed closely with experiment. However, because gas evolution and electrolysis by-products were neglected, the simulated surface reached its final state considerably faster than the real one, and the substrate was stainless steel; the approach therefore demonstrates the methodological value of moving-boundary modeling but does not yet provide validation for evolving Ti surfaces.

Regarding the second question, Wang et al. used a simplified two-dimensional microgroove model to predict the effect of applied voltage on the evolution of the groove-bottom profile in Ti-6Al-4V, and applied the calculated field distributions to guide experiments on grooves of different aspect ratios, ultimately reducing the roughness of both notch and bottom regions from approximately 250 nm to below 40 nm (Figures 8b–d). (Wang et al., 2024) Qin et al. developed a three-dimensional electrochemical–fluid model for support removal from SLM-fabricated Ti-6Al-4V, whose simulation-guided experiments achieved complete removal of the targeted connections with an average difference between simulated and measured material removal of 8.22% (Qin et al., 2025). Both studies show that calibrated models can provide useful guidance for otherwise inaccessible features. In both cases, however, the geometry was idealized—a single cross-section in one, simplified repeating support units in the other—and flow was assumed laminar with bubbles and thermal effects neglected, so the agreement constitutes validation for the investigated configurations rather than universal predictability. For fully interconnected porous AM Ti structures, where narrow pore throats, stagnant electrolyte, trapped gas, and deviations between ominal CAD and as-built geometry complicate the boundary conditions, experimentally validated ECP models remain scarce.

Regarding the third question, Wang et al. calibrated a multiphysics model of Nb cavity electropolishing against coupon-scale polarization measurements and then applied it to the full cavity geometry, identifying a coolant temperature of 7 °C and an intermediate flow rate of 10 gallons per minute (GPM) as favorable conditions for maintaining uniform surface current (Wang et al., 2022). The study demonstrates how coupon-scale measurements of the transport-limited operating window can be transferred into a component-scale model. However, the validation was performed against polarization behavior, whereas the local current-density, temperature, and oxide-thickness distributions within the cavity were numerical predictions on a fixed geometry. A model may therefore reproduce measured polarization behavior while retaining uncertainty in its spatial predictions after transfer to a larger and more complex geometry.

Taken together, these studies indicate that the reliability of an ECP model depends on the quantity and scale at which it has been validated: agreement with polarization curves supports identification of the operating window, whereas agreement with dissolved mass or evolving surface profiles provides stronger evidence for spatial removal predictions, and component-level validation is required before predictions can be generalized across a complex part. At present, simulation is most credible as an experimentally constrained screening and diagnostic tool—identifying regions susceptible to under- or over-polishing, comparing electrode and flow configurations, and narrowing the conditions that must be tested—provided predictions remain within the calibrated ranges of electrolyte, temperature, geometry, and flow. It is not yet a substitute for component-level validation. Numerical and experimental approaches are therefore best used iteratively: experiments supply the parameters that calibrate the model, and the calibrated model guides the selection of subsequent experimental conditions.

## Discussion and conclusion

AM has expanded the use of Ti alloys, but the as-built surface—characterized by balling, partially melted particles, scan-track ridges, and porosity—often requires post-processing. Among the available methods, ECP is particularly attractive for the complex and internal geometries produced by AM. This review has treated ECP not as a catalogue of operating conditions, but as an interfacial design problem. The specimen defines the initial current-distribution problem: the wavelength of its asperities relative to the effective diffusion-layer thickness, δ, influences the rate of Wagner-type amplitude decay described by Equation 4, while curvature and the Wa in Equation 5 determine the extent to which geometry-driven current concentration contributes to leveling. The applied electrochemical parameter then determines whether the process enters the transport-limited polishing region, outside which uniform leveling is generally not sustained.

The electrolyte contributes to this interfacial state through several coupled functions. The solvent influences viscosity and diffusivity; the supporting electrolyte controls conductivity and ohmic losses; acidic or reactive components affect dissolution and interfacial-film stability; and additives modify polarity, complexation, adsorption, and transport. Viscosity, agitation, temperature, and waveform should not be regarded as fully independent variables, but as interacting means of controlling charge transfer, mass transport, and interfacial-film behavior.

Viewed through this framework, the wide range of conditions compiled in Table 4—including acid-based, organic, ionic-liquid, and deep-eutectic electrolytes applied to both AM and wrought substrates—can be interpreted as different routes to establishing a stable polishing interface. This framework also helps explain why reported optimal conditions differ, and sometimes appear contradictory, even among studies employing similar electrolyte systems. The observed position and width of the limiting-current plateau are influenced not only by electrolyte composition but also by cell geometry, inter-electrode distance, temperature, water content, hydrodynamic conditions, and bath aging. Consequently, a voltage located within the effective polishing window in one configuration may correspond to active dissolution, passivation, or oxygen evolution in another. Direct comparison of reported roughness values is also complicated by differences in the initial surface topography, alloy condition, measurement technique, evaluation length, and roughness parameter employed. Thus, many apparent discrepancies among studies may arise from differences in the electrochemical and interfacial conditions actually established, although differences in mechanistic interpretation and experimental methodology cannot be excluded. Accordingly, reporting voltage alone is insufficient to define an optimal electropolishing condition; the corresponding current response, electrolyte history, temperature, hydrodynamic conditions, and initial surface state should also be documented to enable meaningful comparison across studies.

What remains unresolved is the quantitative coupling of these variables in Ti systems. The effective interfacial-layer thickness is rarely measured directly, the relative contributions of salt-film and acceptor-controlled mechanisms remain uncertain, and the transfer of coupon-scale conditions to component-scale geometries still relies heavily on trial and error. Simulation-assisted design, as discussed in Section “Prediction and Optimization of ECP Using Software”, offers a direct route to addressing the final challenge because the primary, secondary, and tertiary current distributions solved numerically are the same distributions that govern polishing uniformity. More standardized reporting of viscosity, conductivity, agitation, temperature, geometry, and electrical conditions would allow the condition database assembled here to be used more predictively rather than only retrospectively.
